# Psychosocial implications of blindness and low vision in students of a school for children with blindness

**DOI:** 10.12669/pjms.322.8737

**Published:** 2016

**Authors:** Rizwan Ishtiaq, Muhammad Hamid Chaudhary, Muhammad Atif Rana, Abdur Rehman Jamil

**Affiliations:** 1 Dr. RizwanIshtiaq, MBBS. Quaid-e-Azam Medical College, Bahawalpur, Pakistan; 2 Dr. Muhammad Hamid Chaudhary, MBBS. Quaid-e-Azam Medical College, Bahawalpur, Pakistan; 3 Dr. Muhammad Atif Rana, MBBS. Quaid-e-Azam Medical College, Bahawalpur, Pakistan; 4 Dr. AbdurRehman Jamil, MBBS. Quaid-e-Azam Medical College, Bahawalpur, Pakistan

**Keywords:** Psychosocial adjustment, Individual differences, Visual impairment & blindness

## Abstract

**Objective:**

To find out the psychosocial implications of blindness and low vision in students of blind school Bahawalpur.

**Methods:**

A cross sectional descriptive study was carried out in Higher Secondary School for blind, Bahawalpur after getting approval from Institutional review board of Quaid-e-Azam Medical College, Bahawalpur and Principal of Blind School, Bahawalpur. Forty willing students filled a customized questionnaire, consisting of questions about logistic variables and questions regarding areas of satisfaction. Statistical analysis was performed using SPSS version 18.

**Results:**

Out of 40, 55% (22/40) of them were found depressed (as assessed through DSM-lV), 50% (20/40) were having difficulty in making new contact but 52.5% (21/40) were satisfied with family care.

**Conclusion:**

Sixty percent (24/40) of blind school children experienced difficulty in their life. This study showed that blindness or low vision does have psychological implications like feeling of guilt, anxiety, sadness & depression.

## INTRODUCTION

Any trauma or ailment leading to blindness causes a drastic change in the quality of life, more importantly, lifestyle and habits resulting in problems associated with psychological adjustments.^1^

World Health Organization (WHO) has proposed the following definition for blindness.” A physical, psychiatric, intellectual or sensory impairment, whether temporary or permanent, provided that it lasts for a significant period of time, that limits the capacity to perform one or more essential activities of daily life and which can be caused or aggravated by economic and social environment”.^2^

Defective vision has been classified into various grades by WHO which are shown in Table-I. Blindness causes restriction in will and ability to move around and an active control over oneself and the surroundings. Hence, making the disability alone a big factor in making people with blindness feel dejected and desolated.^3^

**Table-I:** WHO grading of blindness.

Category	Visual Impairment	Best corrected visual acuity
0	Normal	6/6 to 6/18 i.e. can see 6/18 or better
1	Visual impairment	<6/18 to 6/60 i.e. cannot see 6/18 but can see 6 60.
2	Severe visual impairment	<6/60 to 3/60 i.e. cannot see 6/60 but can see 3/60.
3	Blind	<3/60 to 1/60 i.e. can’t see 3/60 but can see 1/60.
4	Blind	<1/60 to only PL i.e. can’t see 3/60, can see light.
5	Blind	No light perception i.e. cannot see light

There is an awkward moment when sighted people first meet people with blindness. They come across the dilemma, should they greet a person with blindness with a hand shake as they cannot see the extended hand. This creates apprehension of discomfort and clumsiness which their presence imposes on sighted people.

Consistent attempts by sighted people to avoid any citation or references of vision and words of obvious affection and sympathies in their conversations causes people with blindness to avoid social contact, hence leading to a sense of social seclusion from the society.^3^ People with blindness adapt to not using any facial expressions and bodily gestures and therefore fail to develop the ability of using them in their day to day conversations. They are not able to sense the visual cues as to whose turn it is to speak.^4^

People with blindness may suffer from repudiation, umbrage, inferiority complex, anxiety, depression and similar psychological problems because of their incapacity in comparison to healthy people or due to the feeling of low self-esteem.^5^ According to a population-based survey conducted in 1987-1990, by the Ministry of Health of Pakistan and the World Health Organization (WHO), Pakistan has a significant prevalence (1.68%) of blindness^6^ thus it is imperative to study the effects of blindness on school going children in Pakistan.

The present survey is based on a comprehensive study conducted at Blind School Bahawalpur to measure the psychosocial implication among the children with blindness. The rationale of this study was to determine the level of psychological impairment in the children with blindness & to identify the socio demographic characteristics and essential rehabilitation steps that might improve the confidence & level of satisfaction. The study findings will provide knowledge about the quality improvement leading to understanding & identification of the principal for betterment of individual to maximum level of working capacity.

## METHODS

An observational descriptive cross – sectional study was carried out in Blind School Model Town A, Bahawalpur from December 8, 2014 to January 6, 2015 after getting approval from Institutional review board of Quaid-e-Azam Medical College, Bahawalpur and Principal of Blind School, Bahawalpur. Forty willing students present in school were included in the study. Informed consent was taken from guardians/parents of all participating students. A pre-designed custom made questionnaire was used for data collection, to determine the level of depression based on DSM-IV Criteria for Major Depressive Disorder (MDD).^7^ Children were interviewed after explaining the study and taking their verbal consent. Our questionnaire consisted of questions about logistic variables and questions regarding areas of satisfaction relating to time, care provider, facilities & user charges. Statistical analysis was performed using SPSS version 18.

## RESULTS

Out of 40 students, majority 23 (57%) were males and 17(42%) were females. Age distribution showed 16 students were in age group (10-15 years), 13 were in age group 15-20 years and 11 were in age group 21-25. Mean age of students in our study was 17 ± 3 years with maximum age of 22 years and minimum age of 10 years.

Regarding grades of blindness among children, according to WHO grades, 12 (30%) children were of category 1, 18(45%) children were of category 2, 4 (10%) children of category 4 and 6 (15%) patients of category 5. Out of 40 children with blindness, 9 (22%) had increased appetite, 13 (32%) had no change in appetite while 18 (46%) reported decreased appetite. When asked about worthlessness or feeling guilt, 17 (42%) answered yes, 15 (38%) answered to have feeling of worthlessness and guilt sometimes, 8 (20%) answered to have never experienced such feelings. When inquired about difficulty in concentration, 23 (57.5%) replied “yes”, 8 (20 %) had sometimes difficulty and 9 (22.5%) had never experienced difficulty. Regarding changes in sleeping habits, 23 (57.5%) reported difficulty in sleeping and 17 (42%) reported no change in sleeping habits. While academic performance of 26 (65%) was affected and 14 (35%) said it had no effect. According to DSM IV criteria 22(55%) were found to have at least 5 out of 9 symptoms, and hence according to criteria, are suffering from depression. (Table-II)

**Table-II:** Depression among blind students.

Question	Strongly Agree/yes	Slightly Agree/sometimes	Disagree/no
Depression, Hopelessness	22(55%)	8(20%)	10(25%)
Feeling of Little Energy	14(35%)	13(32.5%)	13(32.5%)
Thoughts of Suicide	16(40%)	10(25%)	14(35%)
Decreased appetite	17(42.5 %)	13(32.5%)	10(25%)
Feeling worthlessness	17(42.5%)	15(37.5%)	8(20%)
Change is sleeping habits	23(57.5%)		17(42.5%)
Decreased academic performance	26(65%)		14(35%)

Twenty (50%) students had difficulty in making new contacts, 9(22.35%) had difficulty in making contacts sometimes and 11(27.5%) students never faced any difficulty. As far as satisfaction with family members was concerned, 21(52.5%) students were found satisfied with family members, 10(25%) were satisfied to some extent and 9(22.5%) were not satisfied at all.

Students found difficulty in doing routine works were 9(22.5%), 8(20%) found difficulty to some extent &23(57.5%) were not having difficulty at all. Thirty one (77%) students belong to parents having annual income less than 40,000 and 9 (22%) students had parents with annual income more than 40,000. 35 (87.5%)students belong to rural areas and 5(12.5%) students were from urban area.

## DISCUSSION

Children with impaired vision or blindness exhibit different range of emotional and physical complications. They feel themselves compromised, victim of some sort of evil, exhibit stereotypical behavior such as anxiety, depression or excessive thought. They face difficulty in social interactions and making contacts and thus prefer to live in isolation. Language, motor or cognitive delays have a proportional effect on social competence exhibited by a child.^8^

Majority of students (55%) in our study were found to be depressed. Studies conducted on visually impaired students showed that many students are stumbling socially as compared to students with normal vision.^9^, ^10^ Due to lack of visual input, decreased level of physical activity, perceptions regarding lack of attractiveness &competence, students with visual impairment are more prone to be isolated from students of normal vision.^11^, ^12^ Use of special media to access curriculum by visually impaired children, like large print and braille may lead to development of superiority complex in visually normal students. This superiority complex is partly due to false belief of having greater intelligence than visually impaired students as they may have superior fluency when reading and writing. Visually impaired but not completely blind students may also be reflected to be at a greater risk than those who are blind for poor social knowledge. In a striking study of social and practical knowledge^13^ students with low vision scored considerably lower (mean results at the 25^th^ percentile) than did those who were blind (mean results at the 50th percentile) on the Practical Knowledge Quotient, possibly because the students with low vision were not offered a similar level of verbal support (explanations) as compared to completely blind students.

Most of the children with blindness (78%) play cricket in their free time. This establishes that extra-curricular activities have a positive effect on the students. Majority of our subjects belonged to rural areas. A significant number of our subjects (77%) were of low socio economic status.

QUESTIONNAIRE

1. Do you feel sad, irritable or empty?

2. Do you like to play role in the daily activities of life?

3. Do you feel increase or decrease in your appetite?

4. Do you feel fatigued or loss of energy nearly every day?

5. Do you feel worthlessness or inappropriate guilt of yourself?

6. Do you feel you have diminished ability to think or concentrate?

7. Do you have recurrent suicidal ideation without a specific plan?

8. Do you feel change in your sleeping habits? Please explain.

9. Do you feel like decreased interest or pleasure in most activities?

10. Do you feel yourself preoccupied with unpleasant worries?

11. Explain your family relationships with you?

12. Explain your relationships with peers?

13. Explain your academic performance?

14. Have you ever caused yourself harm?

15. Do you feel difficulty in contacting your peers or colleagues?

16. Do you hesitate in talking to someone because of your disability?

Our results were a bit similar to with the studies done previously.^14^ The subjects in previous studies showed a great extent of social competence, and because of it they were less prone to social isolation than the Pakistani peer. What may be the cause of such differences? Due to the limitations of this study, it is impossible to answer this question. Most of our subjects were satisfied with their living. Most of them have no difficulty in concentration and processing their daily routine activates.

According to our understanding, personality characteristics play much greater role in social development than cultural ones. In spite of good social contacts and family care as demonstrated from our study, children with blindness are found confused and depressed in our part of world especially. This gives us an idea to prioritize a highly individual interventional program for development of social excellence in these children. Culture should have no impact on such program. As compared to earlier study reports, members faced less problems in commencing and continuing collaborations with their peers. It is important to upgrade child’s presence in group communication. It will ensure the positive behavior of peers toward the visual deterioration of child.

### Limitations of the Study

Despite the fact that this study was the first ever study to present details about psychological distress among blind and low vision students in blind school, the study tools unmasked nonspecific nonphysical sufferings only. Due to the cross sectional nature of our research, we were not able to institute free and easy association between loss of vision and psychological pain. Furthermore, another limitation is that we didn’t establish whether the findings were associated with level of blindness or duration of blindness. Further research must be done to establish association of depression with level of blindness, and anxiety associated with depression in students with blindness.

We suggest that mental care should be balanced along with clinical care in order to decrease the distress outcomes. Family support system should be established and special workshops for parents of blind and low vision student should be conducted. Advance studies are compulsory in this regard.
